# A primary human T-cell spectral library to facilitate large scale quantitative T-cell proteomics

**DOI:** 10.1038/s41597-020-00744-3

**Published:** 2020-11-23

**Authors:** Harshi Weerakoon, Jeremy Potriquet, Alok K. Shah, Sarah Reed, Buddhika Jayakody, Charu Kapil, Mukul K. Midha, Robert L. Moritz, Ailin Lepletier, Jason Mulvenna, John J. Miles, Michelle M. Hill

**Affiliations:** 1 QIMR Berghofer Medical Research Institute, Herston, Brisbane, QLD 4006 Australia; 2 School of Biomedical Sciences, The University of Queensland, St Lucia, QLD 4072 Australia; 3 Faculty of Medicine and Allied Sciences, Rajarata University of Sri Lanka, Saliyapura, 50000 Sri Lanka; 4 UQ Centre for Clinical Research, Faculty of Medicine, The University of Queensland, Brisbane, QLD 4006 Australia; 5 Institute for Systems Biology, Seattle, WA 98109 USA; 6 Australian Institute of Tropical Health and Medicine, James Cook University, Cairns, QLD 4878 Australia; 7 Centre for Molecular Therapeutics, James Cook University, Cairns, QLD 4878 Australia; 8 Centre for Tropical Bioinformatics and Molecular Biology, James Cook University, Cairns, QLD 4878 Australia; 9 Present Address: SCIEX Australia Pty Ltd, Mt Waverley, VIC 3149 Australia; 10 Present Address: CSL Limited, 45 Poplar Rd, Parkville, VIC 3052 Australia; 11 Present Address: Institute for Glycomics, Griffith University, Gold Coast, QLD 4222 Australia

**Keywords:** Data acquisition, Lymphocyte activation, Proteomic analysis, Proteomics, Mass spectrometry

## Abstract

Data independent analysis (DIA) exemplified by sequential window acquisition of all theoretical mass spectra (SWATH-MS) provides robust quantitative proteomics data, but the lack of a public primary human T-cell spectral library is a current resource gap. Here, we report the generation of a high-quality spectral library containing data for 4,833 distinct proteins from human T-cells across genetically unrelated donors, covering ~24% proteins of the UniProt/SwissProt reviewed human proteome. SWATH-MS analysis of 18 primary T-cell samples using the new human T-cell spectral library reliably identified and quantified 2,850 proteins at 1% false discovery rate (FDR). In comparison, the larger Pan-human spectral library identified and quantified 2,794 T-cell proteins in the same dataset. As the libraries identified an overlapping set of proteins, combining the two libraries resulted in quantification of 4,078 human T-cell proteins. Collectively, this large data archive will be a useful public resource for human T-cell proteomic studies. The human T-cell library is available at SWATHAtlas and the data are available via ProteomeXchange (PXD019446 and PXD019542) and PeptideAtlas (PASS01587).

## Abstract (summary)

**Table:** 

Measurement(s)	Proteome • protein expression data
Technology Type(s)	Proteomic Profiling • nanoflow liquid chromatography-tandem mass spectrometry • SWATH MS protein profiling assay • Data-Dependent Acquisition • mass spectrometry
Factor Type(s)	time following T-cell activation
Sample Characteristic - Organism	Homo sapiens

Machine-accessible metadata file describing the reported data: 10.6084/m9.figshare.12991619

## Background & Summary

T-cells are the central regulators of adaptive immunity and among the most diverse cellular compartment in human physiology^1^. While advanced transcriptomics, including single cell transcriptomics, is commonly applied to investigate T-cell subsets to infer T-cell function, post-translational modifications (PTMs), splice variants and other downstream modifications regularly occur and must not be overlooked. Accordingly, quantitative T-cell proteomics is an essential tool in understanding the workings of T-cells in health and disease^1–3^. Nanoscale liquid chromatography-coupled tandem mass spectrometry (LC-MS/MS) has become a standard technique for quantitative proteomic studies in human biology and disease, by which traditional data dependent acquisition (DDA) mass spectrometry (MS) proteomics has added significant understanding to the immunological process^4–6^. The stochastic selection of precursor ions has led to large proportion of missing data, which impacts downstream statistical analyses in proteomics annotation, functional interpretation and therapeutic development^7,8^. To overcome these limitations, data-independent acquisition (DIA-MS) was performed to acquire all ions by dividing ion space. The introduction of the sequential window acquisition of all theoretical fragment ion spectra (SWATH-MS) which is a combination of tandem mass spectrometry performed by quadrupole time-of-flight (QTof) instruments, DIA-MS and a peptide-centric data query method, facilitated the reproducible quantification of proteomes to perform high-throughput analysis of immunological samples^9^. This technique is now standardized to obtain highly reproducible data in international laboratories^10^.

Despite broad adoption of SWATH-MS, its application in the field of immunology has been limited, partly due to the absence of publicly available comprehensive spectral libraries for the hundreds of immune cell subsets defined^11^. A Pan-human spectral library covering ~10,000 proteins was reported by Rosenberger *et al*. in 2014^12^, which unfortunately did not include primary human T-cells. Given the role T-cells play in the regulation of immune compartments, tissues, organs and the general homeostasis of human biology^1^, having a high-quality publicly available spectral library of natural human T-cell proteins will reduce resources, time, sample and analysis for future experiments.

To address this important gap in knowledge, we generated a comprehensive spectral library of primary human T-cells from four genetically unrelated individuals in *ex vivo* and *in vitro* stimulated states. The samples were obtained through off-gel fractionation of tryptic digested protein samples. Collectively, a total of 96 samples were analyzed to generate the primary human T-cell spectral library. Using the above described techniques, we could identify more than 4,800 unique proteins. In an experiment to test the performance of the library on SWATH-MS, we reliably identified and quantified 2,850 proteins across 18 primary human T-cell samples. Further, we performed a direct comparison of our purified human T-cell spectral library performance against the Pan-human spectral library which showed only 38% overlap. The combination of the data from two libraries data sources produced 4,078 proteins, which will provide a useful bassline for future T-cell proteomic investigations. Further, to identify the strengths and weaknesses of SWATH-MS compared with traditional DDA-MS based shotgun proteomic method for T-cell proteomics, we compared the label free DDA-MS data acquired on the same set of samples with SWATH-MS data. We identified an improved performance in SWATH-MS in terms of number of quantified proteins and data reproducibility.

## Methods

### Building a primary human T-cell spectral library

#### Human T-cell isolation, activation and phenotyping

Ethics approval was obtained from the human research ethics committee QIMR Berghofer, Brisbane, Queensland, Australia (HREC #P2058). Written informed consent was obtained from all volunteers. Peripheral blood mononuclear cells (PBMCs) were separated from buffy coats obtained from Red Cross Blood Bank, Australia using a Ficoll-Paque Plus (GE Healthcare, USA) gradient centrifugation. These cells were sorted using pan human T-cell magnetic beads (Miltenyi Biotec, Germany) to obtain primary human T-cells with over > 95% purity as confirmed by flow cytometry. Using the separated PBMCs, we prepared one *in vitro* (SpLib_1) activated and three *ex vivo* (SpLib_2, SpLib_3 and SpLib_4) CD3^+^ T-cell samples. CD3/28 Dynabeads (Thermo Fisher, USA) at 1:1 cell to bead ratio in RPMI 1640 medium, 10% foetal calf serum (Gibco, USA) and 50 units/ml penicillin and 50 μg/ml streptomycin (Gibco, USA) to activate CD3^+^ T-cells. These cells were cultured over 7 days at 37 °C in a humidified, 5% CO_2_ incubator. To validate T-cell purity, enriched cells were stained with LIVE/DEAD Fixable Aqua (Life Technologies, USA), CD3-APC-eFluor780 (eBioscience, USA) and a dump panel including CD14^+^ Pacific Blue (BioLegend, USA), CD16-Pacific Blue (BioLegend, USA), and CD19-Pacific Blue (BioLegend, USA). Only live single cells (excluding doublets) were gated and run on a LSR Fortessa 4 (BD Biosciences, USA). Flow cytometry data was analyzed using FlowJo version 10 (TreeStar, USA).

#### Proteomic sample preparation

**Fig. 1:**
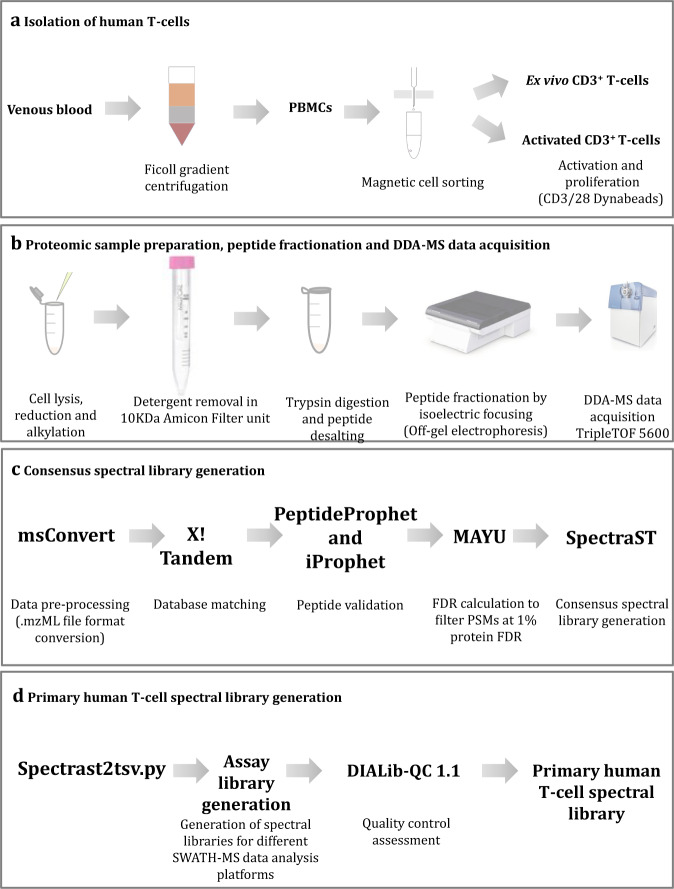
Primary human T-cell spectral library generation workflow. Main steps used in (**a**) isolation of human peripheral blood mononuclear cells (PBMCs), purification of *ex vivo* CD3^+^ T-cells and *in vitro* activation (three *ex vivo* CD3^+^ T-cell samples and one *in vitro* activated CD3^+^ T-cell sample were obtained from PBMCs extracted from four different buffy coats). **(b)** Proteomic sample preparation, peptide fractionation and LC-MS/MS data acquisition. **(c)** Generation of individual spectral libraries for separate DDA-MS dataset. **(d)** Generation of a combined assay library compatible for multiple SWATH-MS data analysis platforms and quality control of assay libraries.

The workflow for primary human T-cell spectral library generation is summarized in Fig. 1. T-cells isolated for the spectral library generation were lysed in 100 µL of buffer composed of 100 mM TEAB (Triethylammonium bicarbonate) (Sigma, USA), 1% sodium dodecyl sulfate (SDS) (Bio-Rad laboratories, USA), 5 mM MgCl_2_ (Sigma, USA) supplemented with 1x Roche complete protease inhibitors (Sigma, USA). Immediately after the lysis, ultrapure benzonase (Sigma, USA) in 50 µL of 50 mM Tris, 2 µL of 20 mM NaCl and 2 mM MgCl_2_ at 1 unit/µl concentration was added to degrade DNA and RNA. The samples were incubated at 4 °C for 45 minutes with constant agitation and the protein concentration was determined using the Pierce BCA assay kit (Thermo Fisher Scientific, USA) following the manufacturer’s protocol. An aliquot of ~600 μg of protein was reduced in 20 mM dithiothreitol (DTT) (Sigma, USA) at 75 °C for 10 minutes. After cooling the sample for ~10 minutes at room temperature, 0.5 M 2-iodoacetamide (IAA) (Sigma, USA) was added for a final concentration of 40 mM, and kept for 30 minutes in the dark at room temperature to alkylate proteins. Detergent removal was performed by the filter aided sample preparation method^13^ using 10 mL, 10 KDa Amicon molecular weight cutoff filter tubes (Merk Millipore, USA). First, the tube was equilibrated by spinning 600 µL of 100 mM TEAB at 3,100 × g for 10 minutes in a 5810 R Eppendorf centrifuge (Eppendorf, Germany). Second, the protein sample was mixed with six volume of freshly prepared 8 M urea (Sigma, USA) in 100 mM TEAB, 10% isopropanol (Sigma, USA) and transferred to the filter unit and spun at 3,100 × g for 30 minutes. Third, detergents were removed using buffer exchange in which the protein mixture was washed with 500 µL of 8 M urea in 100 mM TEAB, 10% (v/v) isopropanol. This was followed by another wash with 500 µL of 100 mM TEAB, 10% isopropanol and then two washes with 500 µL of 50 mM TEAB. In all the washes, samples were spun at 3,100 × g for 30 minutes to remove the detergents. The concentrated protein solution was then collected from the filter unit and transferred to an Eppendorf microcentrifuge tube (Eppendorf, Germany) to perform the protein digestion. Sequencing grade modified trypsin (Sigma, USA) at protein to trypsin ratio of 50:1 was added and incubated overnight at 37 °C in a humidified incubator. The resulting peptides were desalted using a Sep-Pak Vac C18 cartridge (Waters, USA), lyophilized with a speed vacuum prior to fractionation in Agilent 3100 off-gel fractionator (Agilent technologies, USA) using a 24 cm, pH 3–10 IPG strip (GE Health Care, USA) according to the manufacturer’s protocol. During fractionation, a maximum current of 50 µA until 50 kV/h was used. Each fraction was lyophilized and then resuspended in 30 µL of MS grade water with 2% acetonitrile (Sigma, USA), 0.1% formic acid (v/v) (Sigma, USA) prior to LC-MS/MS analysis.

#### DDA-MS data acquisition

Peptide samples resuspended in 30 µL of MS grade water with 2% acetonitrile, 0.1% formic acid (v/v) were spiked with the indexed retention time (iRT) peptides (1:100) (Biognosys, Switzerland). To chromatographically separate the peptide samples, we used either Eksigent cHiPLCTM-nanoflex (For SpLib_1, SpLib_1 and SpLib_3) or Eksigent Nano Ultra 1D + (SpLib_4) systems (SCIEX, USA), where 10 uL of each samples was injected to a 15 cm long ChromXP C18-CL column (particle size 3 µm, 120 Å, 150 mm × 0.075 mm). A pre-concentration step (10 minutes) was performed employing a ChromXP trap column (C18-CL, 5 µm, 120 Å, 0.3 × 10 mm) before commencement of the gradient. A flow rate of 500 nL/minute was used to generate SPLib_1, SPLib_2 and SPLib_3, while 250 nL/minute flow rate was used for SPLib_4. The mobile phase consisted of solvent A (0.1% formic acid/H_2_O) and solvent B (100 acetonitrile/0.1% formic acid) was used for the three consecutive linear gradients for peptide elution: 5–10% solvent B (acetonitrile/0.1% formic acid) over 2 minutes, 10–40% solvent B over 58 minutes and 40–50% solvent B over 5 minutes. A final gradient from 50% to 95% solvent B in 10 minutes was used to clean the column. Eluates from the column were directly injected into the NanoSpray II ionization source of a TripleTOF 5600 MS/MS System (SCIEX, USA) operated in positive ion electrospray mode. The peptides and the DDA-MS acquisition protocol were created using Analyst 1.5.1 software (SCIEX, USA) in which a 300–2000 (*m/z*) precursor (MS) and 100–2000 (*m/z*) product (MS/MS) mass ranges were used. To acquire DDA-MS data for SPLib_1 to 3, we used 250 and 100 millisecond MS and MS/MS accumulation times respectively and the ions observed in the TOF-MS scan exceeding a threshold of 50 counts and a charge state of + 2 to + 4 were set to trigger the acquisition of MS spectra for a maximum of 10 most intense ions. DDA-MS data for SpLib_4 were acquired using 250 and 150 millisecond MS and MS/MS accumulation times respectively. Ions within the charge status of + 2 to + 5 were used to trigger the acquisition of MS spectra for a maximum of 12 most intense ions.

#### Human T-cell spectral library generation

**Fig. 2:**
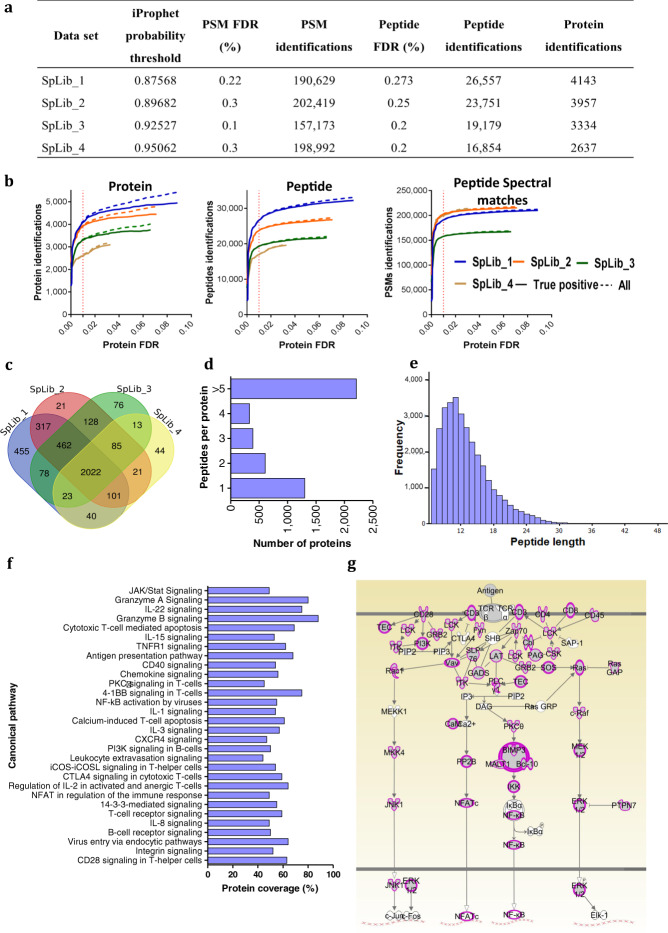
Statistics of the primary human T-cell spectral library. (**a**) Statistics of individual spectral libraries used in the generation of human T-cell spectral library. **(b**) Protein, peptide and peptide spectral matches (PSMs) of individual libraries as function of protein FDR. **(c)** Venn diagram depicting the protein overlaps between different spectral libraries. **(d**) Frequency distribution of peptides of different lengths in human T-cell spectral library. (**e**) Frequency distribution of proteins with different peptide numbers in human T-cell spectral library. **(f**) First 30 significant immune related canonical pathways represented by the T-cell spectral library (Core analysis, Ingenuity pathway analysis, Qiagen Bioinformatics, USA) **(g**) Protein coverage of the human T-cell spectral library for T-cell receptor (TCR) signaling pathways. Proteins highlighted in pink are included in human T-cell spectral library. (SpLib_1 - *in vitro* activated human T-cell spectral library, SpLib_2, 3 and 4 - *ex vivo* human T-cell spectral libraries).

Acquired DDA-MS data was analysed through Trans-Proteomic Pipeline (TPP)^14^ to generate the primary human T-cell spectral library. In brief, after converting DDA-MS data into mzML file format (msConvert, ProteoWizard), each data set was searched using X!Tandem with k-score plugin and Comet against UniProtKB/Swiss-Prot human reviewed database (downloaded on 21.11.2017) with added decoy database and iRT sequence. Search parameters were set as; parent mass error of ± 50 ppm, fragment mass error of ± 0.05 Da, trypsin digestion allowing two missed cleavages while carbamidomethyl (57.021464@C) and oxidation (15.994915@M) were included as fixed and variable modifications respectively. Results were then scored and combined with PeptideProphet^15^ and iProphet^16^, respectively to proceed with the false discovery rate (FDR) calculation using MAYU^17^. Data filtered through the iProphet probability cut-off corresponding to protein FDR at 1% (Fig. 2a) as calculated by MAYU and spectraST^18^ were then used to generate individual spectral libraries normalised to iRT peptides. Then a consensus spectral library was built using the “Consensus” build action with “Union” join option in the SpectraST^18^. We used spectrast2tsv (msproteomicstools 0.8.0) to generate the final assay library compatible with SWATH-MS windows from 300–2000 *m/z* mass range and for different SWATH-MS data analysis platforms. DIALib-QC v1.1 was used to assess the quality of these spectral libraries and to validate for theoretical *m/z* values correctness of both precursor and fragments ions in the libraries^19^. Decoy sequences were added to OpenSWATH assay library using OpenSWATH decoy generator^20^.

### Validation of the human T-cell spectral library

#### Isolation and activation of human CD4^+^ T-cells

**Fig. 3:**
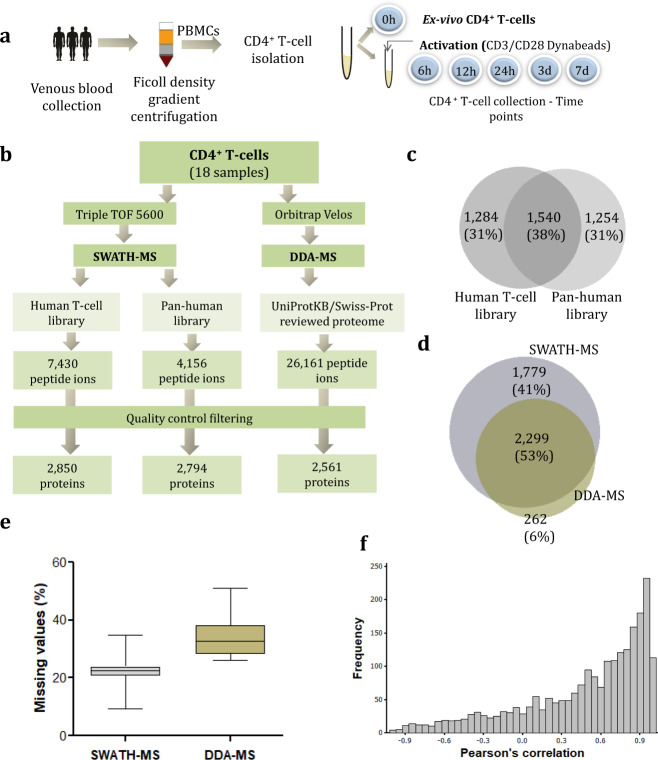
SWATH-MS data analysis against human T-cell and Pan human libraries and comparison with DDA-MS data. (**a**) Experimental protocol used to obtain human CD4^+^ T-cells at different *in vitro* activated status. Samples were obtained from three different donors. **(b)** Number of peptides and proteins detected by the analysis of CD4^+^ T-cells by SWATH-MS against human T-cell and pan-human spectral libraries and DDA-MS. **(c**) Proportional Venn diagram depicting the overlap between quantified SWATH-MS proteins against the two spectral libraries. **(d**) Proportional Venn diagram depicting the overlap between all the quantified SWATH-MS proteins and DDA-MS proteins. **(e)** Box plots showing the missing peptide intensity values in SWATH-MS and DDA-MS data (central lines and boxes represent means and 95% confidence intervals respectively while whiskers are 2.5 to 97.5 percentiles). **(f)** Distribution of Pearson correlation coefficient between protein expression data obtained by SWATH-MS and DDA-MS for human CD4^+^ T-cell time series experiment.

We validated our human T-cell spectral library using a set of SWATH-MS and DDA-MS data generated from a time series T-cell proteomic experiment (Fig. 3a). CD3^+^ T-cells were isolated from PBMCs obtained from three volunteer blood donors following the procedure mentioned above. These cells were further purified using human CD4^+^ T-cell magnetic beads (Miltenyi Biotech, Germany) to obtain untouched CD4^+^ T-cells with over 95% purity, as assessed by flow cytometry. An aliquot from each donor was taken as *ex vivo* (0 hour) sample. The remaining purified cells from each donor were *in vitro* activated and cultured using the protocol above and harvested at 6 hours, 12 hours, 24 hours, 3 days and 7 days of post activation. Collected cells were washed three times with PBS and the cell pellets stored at −80 °C for batch proteomics processing.

#### Preparation of samples for time series T-cell proteomic experiment

T-cell pellets obtained from time series experiment containing ~1 × 10^6^ CD4^+^ T-cells were thawed on ice, and lysed in 2% SDS in 100 mM TEAB in the presence of protease inhibitor cocktail. After assessing the protein quantity using pierce BCA protein assay kit, ~20 μg from each cell lysate was separated for reduction (10 mM tris(2-carboxyethyl)phosphine/TCEP at 60 °C for 30 minutes) and alkylation (40 mM 2-Chloroacetamide/2CAA at 37 °C in dark for 30 minutes). Protein co-precipitation with trypsin in cold (−20 °C) 100% methanol^21^ was used to obtain detergent free protein pellets. These protein pellets were washed sequentially using 90% and 100% methanol and digested overnight with sequencing grade modified trypsin with final trypsin: protein ratio of 1:50 in 50 μl of 50 mM TEAB. StrataX C18 solid phase extraction 1.3 mL columns (Phenomenex, USA) were used to desalt peptides and microBCA (Thermo Fisher scientific, USA) protein assay was used to quantify the peptide concentration.

#### Acquisition of SWATH-MS and DDA-MS data

Peptide samples were resuspended in 10 µL of MS grade water with 2% acetonitrile, 0.1% formic acid (v/v) and spiked with the indexed retention time (iRT) peptides (1:100). For SWATH-MS data acquisition, ~1 µg of peptides from each sample was injected at a flow rate of 250 nL/minute into Eksigent Nano Ultra 1D + system. A ChromXP C18-CL Trap column (C18-CL, 5 µm, 120 Å, 0.3 × 10 mm) and a ChromXP C18-CL column (particle size 3 µm, 120 Å, 150 mm × 0.075 mm) were used to separate peptides in the same chromatographic gradient described above. Using 100-variable window method, the eluted peptides were analyzed over 400–1250 *m/z* mass range by the same mass spectrometer to obtain the SWATH-MS data. Both MS and MS/MS data were accumulated over a period of 25 milliseconds at total cycle time of 2.572 seconds. The variable window method was generated according to the manufacturer’s instructions using Analyst 1.7 TF software (SCIEX, USA).

For DDA-MS data acquisition, ~1 μg of peptides from each sample was injected to ProteCol C18 trap column in Prominence Nano LC system (Shimadzu, Japan) to separate the ions in a ProteCol C18 (200 Å, 3 μm particle size, 150 mm × 150 μm) column at a flow rate of 1 μl/minute over 180 minutes linear gradient. The mobile phase consisted of solvent A (0.1% formic acid) and solvent B (100 acetonitrile/0.1% formic acid) was used for the three consecutive linear gradients for peptide elution: 5–10% solvent B (acetonitrile/0.1% formic acid) over 5 minutes, 10–27% solvent B over 147 minutes and 27–40% solvent B over 10 minutes. A final gradient from 40% to 95% solvent B in 10 minute was used to clean the column. The Nano LC system was operated with the Chromeleon software (version 6.8, Dionex) embedded in Xcalibur software (version 3.0.63, Thermo Fisher Scientific, USA). Eluted peptides were ionized using Nano spray (Thermo Fisher Scientific, USA) ion source (ion spray voltage - 1.75 V, heating temperature 285 °C). Peptide ions separated by Nano LC system were analyzed using a Velos Pro Orbitrap mass spectrometer (Thermo Fisher Scientific, USA). In DDA-MS, the MS was controlled and operated using the Xcalibur software to obtain MS and MS/MS spectral data for maximum of 15 peptide ions with charge status between +2 to +4 at 1.96 second window time. Collision-induced dissociation mode was used to fragment the peptides in the ion trap.

#### SWATH-MS data analysis

PeakView v2.2.0 (SCIEX, USA) with the SWATH-MS acquisition MicroApp 2.0.1.2133 was used to extract SWATH-MS peak areas. Ion library parameters were set with 6 peptides per protein, 6 transitions per peptides, peptide confidence threshold of 99% and FDR threshold of 1%. The time window and width were set to 3 minute and 75 ppm, respectively in XIC manager. The data were analysed separately against both primary human T-cell spectral library and Pan-human spectral library reported by Rosenberger *et al*. in 2014^22^. In the PeakView analysis, retention times for all SWATH-MS experiments were auto-recalibrated based on iRT peptide retention times. Quantitation table files for fragment ions, peptides and proteins were generated using PeakView and MarkerView v1.2.1.1 (SCIEX, USA) and the peptides quantified with peak areas of minimum two fragment ions were selected to obtain the area under the curves of peptides to calculate the relative intensities of proteins for normalization and differential expression (DE) analysis.

#### DDA-MS data analysis

DDA-MS data were analyzed against UniProtKB/Swiss-Prot reviewed human proteome database using MaxQuant (Release 1.6.0.16) software^23^. In the analysis, precursor and product mass tolerance were set as ± 20 ppm and ± 40 ppm respectively to identify the peptides up to maximum charge of + 7 at one miscleavage. Based on unique and razor peptide intensities, proteins at 1% FDR were quantified between runs which allowed the maximum number of peptide identifications.

#### Statistical analysis

Protein intensities across different samples were normalized using variance stabilizing normalization-global (VSNG, R language) method. Figures and statistical analysis were generated with R language and GraphPad Prism (version 8, GraphPad Software, USA) graphical package, and Microsoft Excel software packages (Microsoft, 2010). Analysis of pathway over-representation was performed using ingenuity pathway core analysis (IPA, Qiagen Bioinformatics, USA). In MaxQuant analysis, proteins with multiple UniProtKB/SwissProt protein accessions and m-score less than 5 were excluded to increase the precision of the quantification data. Moreover, in both datasets, proteins which did not have intensity values for either >50% of the samples (n > 9) or two out of three replicates at a given time point were removed from the further analysis. Missing values in the remaining proteins were imputed using maximum likelihood algorithm (Bioconductor, R language). Differential expression of these proteins were calculated in terms of log_2_ fold change in which protein intensity data at each time point following activation were compared with that of *ex vivo* (0 h) value.

## Data Records

All the raw data (DDA), peak (mzML) and search result (pepXML) files^24^ used to generate the human T-cell spectral library, and the consensus spectral library have been deposited in the ProteomeXchange Consortium through the PRIDE partner repository^25^. Primary human T-cell spectral library^26^ generated for different SWATH-MS data analysis platforms and their quality control reports generated through DIALib-QC v1.1 have been made available via the PeptideAtlas (SWATHAtlas) repository.

The raw data (SWATH-MS) files generated from the human CD4^+^ T-cell samples obtained at different activation stages, and PeakView search results and quantification data^27^ have been deposited in the ProteomeXchange Consortium through the PRIDE partner repository^25^.

## Technical Validation

### Filtering high quality spectral data

To reduce false-positive identification, we used Mayu^17^ to calculate the FDR at protein level. We observed high quality spectral data as assessed by the number of total and true positive peptide spectral matches (PSMs), and the peptides and proteins detected as a function of protein FDR. In all four datasets, the number of identified proteins was saturated at 5% of protein FDR and the PSMs and peptides included in spectral libraries had FDRs considerably less than 1% (Fig. 2a,b).

### Primary human T-cell spectral library for SWATH-MS

For SWATH-MS, we used four different sets of off-gel fractionated primary human T-cells to increase the proteins and peptide variability of the final spectral library. This resulted in detection of over 2,500 true positive proteins from each dataset filtered at 1% protein FDR and 2,022 proteins were common to all four libraries (Fig. 2c). Next, we combined all the individual spectral libraries using spectrastST^18^, and used spectrast2tsv to generate the ‘Primary human T-cell spectral library’ containing 232,038 high quality transitions for 31,870 peptides of 4,833 distinct proteins. Over 45% of these proteins were identified with 5 or more peptides (Fig. 2d). Even though there were ~25% of the proteins identified with single peptide hits, high quality fragment ion spectra can be assured due to the selection of peptide spectral matches at 1% protein FDR (Fig. 2a). The number of amino acids in these peptides was varied between 7- 49 with mean of 13 (±4.48) (Fig. 2e). Moreover, we identified >50% of protein coverage for major immune pathways in IPA canonical pathways (Fig. 2e,f).

### Comparison of SWATH-MS and DDA-MS data

Across the 18 samples, SWATH-MS data analysis against human T-cell library quantified 7,430 peptides (~27% of the peptides of the spectral library) including 6 iRT peptides corresponding to 3,005 proteins (Fig. 3b) at 1% protein FDR. Quality control filtering to remove less reproducible proteins was resulted in accurate quantification of 2,850 proteins across 18 samples. Even though the Pan-human library comprised spectral data for larger number of peptides, the analysis resulted in less peptide coverage compared to the human T-cell library, with only 4,156 peptides at 1% FDR corresponding to 2,794 proteins (Fig. 3b). Interestingly, there was only 38% protein overlap between the SWATH-MS data obtained from two libraries (Fig. 3c). These overlapping, but two distinct groups of proteins were resulted in quantification of 4,078 proteins in CD4^+^ T-cells across 18 samples (Fig. 3c). Collectively, analysis of the SWATH-MS dataset against both libraries improved the number of quantified proteins detected by 45% and thus the two libraries can be considered as complementary.

In contrast to SWATH-MS, DDA-MS analysis quantified 3,531 proteins at 1% protein and peptide FDR. However, quality control filtering resulted in reduction to 2,562 high quality proteins. Only 262 new proteins were quantified by DDA-MS compared to the sum of proteins quantified by SWATH-MS (Fig. 3d). As expected, SWATH-MS returned less missing data compared to DDA-MS, particularly at the peptide level (Fig. 3e). Further, we calculated the correlation coefficient values between DE data of SWATH-MS and DDA-MS using Pearson’s correlation. Over ~70% of proteins showed a correlation coefficient over 0.3, indicating a considerably high consistency between the two data acquisition methods (Fig. 3f). Importantly, SWATH-MS achieved an improved depth of protein quantification with half the LC-MS/MS run time which is a crucial factor in terms of cost and robustness of sample analysis.

## Usage notes

While peptide samples for spectral library generation were prepared using filter aided sample preparation method in which trypsin was used for protein digestion, this library is suitable for accurate quantification of SWATH-MS data generated from the samples prepared from any other protein lysis protocols which uses trypsin, with/without LysC for complete digestion along with reduction and alkylation of cysteine bonds. This was exemplified by our use of a methanol co-precipitation procedure when preparing the time series human T-cell proteomic samples to validate the human T-cell spectral library.

### Compatibility of spectral library to other LC-MS/MS settings

In this study, we used a TripleTOF 5600 mass spectrometer and three consecutive linear gradients over 90 minute to acquire DDA-MS data for spectral library generation. As the library contains spectral data for iRT peptides, retention times can be re-aligned and thus, can be used in analyzing the data acquired with different gradients^28^.

## Data Availability

A code used to generate the primary human T-cell SWATH spectral library building using the TPP is described in a recent publication^14^.
